# Supplementary material for: Insulin resistance assessed by short insulin tolerance test and its association with obesity and insulin resistance-related parameters in humans: A pilot randomized trial
Source: PLoS One. 2024 Jun 21;19(6):e0297718. doi: 10.1371/journal.pone.0297718 (PMC11192359; doi:10.1371/journal.pone.0297718)
Supplement: S1 Protocol — (DOCX) [file pone.0297718.s002.docx]

Independent clinical research

**Insulin resistance assessed by short insulin tolerance test and its association with obesity and insulin resistance-related parameters in humans**

Research implementation plan

Research director　 Taku Watanabe

Department of Respiratory Medicine, Faculty of Medicine,

Hokkaido University

Date of creation

First edition March 29, 2016

Revision May 30, 2016

July 1, 2016

August 24,2016

September 12.2016

Table of contents

1. Research background  ………………………………………………………………3

2. Research purpose ……………………………………………………………………3

3. Overview of the study drug …………………………………………………………3

4. Target audience and eligibility criteria …………………………………………………4

5. Research method ………………………………………………………………………6

6. Case registration and allocation method …………………………………………………10

7. Observation and inspection items …………………………………………………………11

8. Expected benefits and disadvantages (burdens and risks) …………………………………12

9. Evaluation item ……………………………………………………………………………14

10. Discontinuation criteria for individual research subjects and responses after research implementation ………………………………………………………………………………14

11. Handling of test results obtained from research on individual research subjects …………15

12. Handling when an adverse event occurs …………………………………………………15

13. Approval, changes, and revisions of research implementation plans, etc. ………………16

14. Discontinuation, interruption, and termination of research ………………………………16

15. Research implementation period …………………………………………………………17

16. Target number of cases, basis for setting it, and statistical analysis method ………………17

17. Consideration for the human rights of research subjects …………………………………18

18. Handling of personal information ………………………………………………………18

19. How to obtain consent ……………………………………………………………………18

20. Response to and compensation for health damage to research subjects …………………20

21. Contents and method of reporting to the head of the research institution ………………20

22. Efficacy and Safety Evaluation Committee ……………………………………………… 21

23. Cost burden for research subjects …………………………………………………………21

24. Methods of storage and disposal of samples, information, etc. ……………………………21

25. Methods of disclosing information regarding research and publication of research results22

26. Regarding ownership of intellectual property rights……………………………………22

27. Research funding and conflicts of interest ………………………………………………23

28. monitoring ………………………………………………………………………………23

29. audit ……………………………………………………………………………………23

30. Research implementation structure ……………………………………………………23

31. Reference materials/literature list ……………………………………………………24

1. Research background

Diabetes is a disease caused by a complex interplay between decreased insulin secretion from pancreatic β cells and insulin resistance in target organs, and it is important to accurately evaluate both in order to understand its pathology. Among the methods for evaluating insulin resistance, the glucose clamp method¹⁾ and the minimal model method¹⁾ are accurate, but the methods are complicated and place a heavy burden on the subject, making them difficult to perform routinely in daily clinical practice. On the other hand, the HOMA method is often used in daily clinical practice because of its simplicity as it can be calculated only from fasting blood glucose levels and blood insulin levels, but it is used in patients with fasting blood glucose levels of 140 mg/dl or higher, who have a strong decrease in insulin secretion, or who are undergoing insulin treatment. It also has the disadvantage of being inaccurate as an indicator for patients on insulin treatment²⁾. In addition, an insulin tolerance test was reported by Rabinowitz and Zierler³⁾ in 1962 as a relatively simple method for evaluating insulin resistance; catecholamines, GH, cortisol, and other insulin antagonistic hormones, which can affect the results, and have recently become obsolete. Later, a short-term insulin tolerance test (SITT) was proposed by Bonora et al.⁴⁾, which improved the shortcomings of the conventional insulin tolerance test such as hypoglycemia, and it became used as an indicator of insulin resistance in clinical practice and in clinical papers.

We take advantage of the insulin tolerance test/SITT method, which can accurately assess insulin resistance without being affected by treatment details or blood glucose levels, and we offer subcutaneous insulin injection, which is simpler, less invasive, and has a lower risk of hypoglycemia. We devised an insulin tolerance test with subcutaneous insulin injection(the simple insulin tolerance test) to evaluate insulin resistance.

In this study, we will examine the usefulness of a simple insulin tolerance test by comparing it with the SITT method to evaluate insulin resistance. Furthermore, based on the results of evaluation using the SITT method and the simple method, we will also examine the relationship between insulin resistance and the maximum amount of insulin used (Study 1).

Furthermore, after completing Study 1 and evaluating the usefulness of the simple insulin tolerance test, we will also evaluate the reproducibility of this test (Study 2).

1. Purpose of the research

We will perform a simple insulin tolerance test and SITT method on diabetic patients admitted to our department for educational purposes, and examine whether insulin resistance can be evaluated using a simple insulin tolerance test. Furthermore, we will examine the relationship between the maximum amount of insulin used to achieve good glycemic control and insulin resistance. In addition, in order to stratify insulin resistance based on the presence or absence of glucose intolerance, the simple insulin tolerance test and SITT method will be administered to volunteers who have not been diagnosed with diabetes in the past and compared.

1. Overview of study drug HumulinⓇR

·Generic name: Insulin human (genetically recombinant) injection

·Prescription drugs listed in drug prices (December 2008)

·Manufacturer/distributor: Eli Lilly Japan Co., Ltd.

·Mechanism of action: Binds to insulin receptors expressed in the liver, skeletal muscles, and fat, exerting a hypoglycemic effect

·Indication: Diabetes for which insulin therapy is indicated

·Dosage and administration: In adults, 4 to 20 units are usually injected subcutaneously at a time before each meal, but the number of doses may be increased or other insulin preparations may be administered in combination. Thereafter, the dosage will be increased or decreased depending on symptoms and test findings, but the maintenance dose is usually 4 to 100 units per day for adults. However, the above dosage may be exceeded if necessary. For diabetic coma, administer subcutaneous, intramuscular, intravenous injection, or continuous intravenous infusion as necessary.

·Adverse events: Side effects were reported in 27 (1.89%) of the 1428 patients evaluated for safety at the time of approval, the main being injection site disorders in 13 (0.91%) and AST/ALT elevation in 4 (0.28%). The side effects, including abnormal changes in laboratory test values, were reported in 494 (10.77%) of the 4,588 cases evaluated for safety in the drug use results survey, and the main cause was metabolic and nutritional disorders such as hypoglycemia in 455 cases (9.77%).

・Clinical use results: Summary of results from clinical trials conducted on diabetic patients who require insulin administration (n=124). Average HbA1c: 1-2 months: -0.09%, 3-4 months: +0.05%, 5-6 months: +0.21%. *HbA1c value is a value measured before it was standardized as JDS value/NGSP value.

·Drug price: 330 yen/ml

*Please refer to the attached document for details.

**Please refer to the package insert [Pharmacokinetics] for the changes in blood glucose levels when 0.1 unit/kg of this drug is subcutaneously injected into healthy adults.

1. Target audience and eligibility criteria

(1) Among the subjects, those who (2) meet all the inclusion criteria and (3) do not fall under any of the exclusion criteria will be eligible.

1. Target audience Diabetic patients admitted to the Department of Internal Medicine I, Hokkaido University Hospital, and volunteers with no previous history of diabetes or hypoglycemic diseases. In order to study the usefulness of this method as an evaluation method for insulin resistance, we will target diabetic patients and volunteers with different degrees of insulin resistance.
2. Selection criteria (inpatients)
3. Persons who are 20 years of age or older at the time of obtaining consent
4. Diabetic patients with early morning fasting blood glucose of 140 mg/dl or more after blood collection on the day after hospitalization (or on the weekday after the holiday if the day falls on a holiday)

③Patients who have received sufficient explanation and informed written consent of their own free will to participate in this study.

Selection criteria (volunteer)

- 1. Those who are 30 years of age or older and have a BMI of 18.5 kg/㎡ or more at the time of obtaining consent
  2. Those who have not been diagnosed with diabetes or a disease that causes hypoglycemia in the past.
  3. Those who have given their free written consent after receiving sufficient explanation and understanding to participate in this research.

1. Exclusion criteria (inpatients)
2. Those whose diabetic retinopathy condition is not stable
3. People with diabetic nephropathy stage 4 or higher
4. Persons with untreated ischemic heart disease
5. Epilepsy patients
6. Subjects with a history of hypersensitivity to the ingredients of the drug used in this study
7. Pregnant women, lactating women, or those who may be pregnant
8. Any other person who is judged by the research director or co-researcher to be inappropriate as a research subject.

Exclusion criteria (volunteer)

1. Those who have been diagnosed with a disease that causes hypoglycemia (blood glucose less than 60 mg/dl) in the past.
2. Subjects with a history of hypersensitivity to the ingredients of the drug used in this study
3. Pregnant women, lactating women, or those who may be pregnant.
4. Those under 30 years of age and with a BMI of less than 18.5 kg/㎡
5. Those with untreated ischemic heart disease
6. Epilepsy patients
7. Any other person who is judged by the research director or co-researcher to be inappropriate as a research subject.

4) How to recruit volunteers

The attached recruitment materials will be posted inside Hokkaido University and volunteers will be solicited.

5. Research method

(1) Type and design of research

Confirmatory clinical trial

(2) Exam outline

After completing all of Study 1, statistically analyze the clinical data obtained in Study 1, and if the usefulness of the simple insulin tolerance test is suggested, conduct Study 2 to verify the reproducibility of the simple insulin tolerance test. Inpatients will participate in either Study 1 or Study 2. Volunteers will be able to participate in both studies.

<Research 1>

Dietary therapy (25-30 Kcal/normal body weight) among diabetic patients admitted to the Department of Internal Medicine I, Hokkaido University Hospital SITT and a simple insulin tolerance test were conducted on separate days (within 3 days) for patients whose blood glucose was 140 mg/dl or more in the early morning fasting blood sample on the day after admission (or on a weekday after the holiday in the case of holidays) to be done. Calculate the rate of disappearance of plasma glucose concentration (Kitt) from the decrease curve of plasma glucose concentration. The smaller the Kitt value, the stronger the insulin resistance. In addition, we measured the maximum amount of insulin required to achieve good blood glucose control (early morning fasting blood glucose less than 130 mg/dl, 2-hour postprandial blood glucose less than 180 mg/dl), measured body composition and basal metabolism using impedance methods, We measure body fat area and muscle mass at the height of the navel using CT, evaluate non-alcoholic fatty liver disease, and measure waist-to-hip ratio. Combining these results with the results of a simple insulin tolerance test, we will examine its usefulness as an insulin resistance evaluation method.

Total amount of insulin required to relieve glucotoxicity

SITT or simple insulin tolerance test

SITT or simple insulin tolerance test

Obtaining eligibility

Obtaining consent

CT, body composition/basal metabolism measurement (during hospitalization period)

Patient background,

blood pressure, pulse, blood sampling, urine test

CGM started

プラセボ1回1錠1日1回朝食後服用

入院

(2週間)

Length of hospital stay (about 1 month)

Both tests will be done within 3 days. The order of SITT and simple insulin tolerance test will be randomly assigned.

②Conduct the SITT and simple insulin tolerance test on volunteers on separate days (within 3 days). Calculate the rate of disappearance of plasma glucose concentration (Kitt) from the decrease curve of plasma glucose concentration. The smaller the Kitt value, the stronger the insulin resistance. Furthermore, we will measure body composition, basal metabolism, and waist-hip ratio using the impedance method, and together with the results of a simple insulin tolerance test, we will examine its usefulness as a method for evaluating insulin resistance.

SITT or simple insulin tolerance test

SITT or simple insulin tolerance test

Obtaining eligibility

Obtaining consent

blood sampling,

body composition/basal metabolism measurement

Patient background,

blood pressure, pulse

プラセボ1回1錠1日1回朝食後服用

入院

(2週間)

Both tests will be done within 3 days. The order of SITT and simple insulin tolerance test will be randomly assigned.

<Research 2>

- 1. Among diabetic patients hospitalized in the Department of Internal Medicine I, Hokkaido University Hospital, with dietary therapy (25-30 Kcal/normal body weight), blood glucose level was 140 mg/day in the early morning fasting blood sample on the day after admission (or on a weekday after holidays in the case of holidays). Perform a simple insulin tolerance test twice on different days (within 3 days) for patients with DL or higher to confirm the reproducibility of the simple insulin tolerance test.

simple insulin tolerance test①

simple insulin tolerance test②

Obtaining eligibility

Obtaining consent

CT, body composition/basal metabolism measurement (during hospitalization period)

CGM started

Patient background,

blood pressure, pulse, blood sampling, urine test

プラセボ1回1錠1日1回朝食後服用

入院

(2週間)

Both tests will be done within 3 days.

Length of hospital stay (about 1 month)

- 1. Confirm the reproducibility of the simple insulin tolerance test by conducting a simple insulin tolerance test on volunteers twice on different days (within 3 days).

simple insulin tolerance test②

simple insulin tolerance test①

Obtaining eligibility

Obtaining consent

Both tests will be done within 3 days.

blood sampling,

body composition/basal metabolism

Patient background,

blood pressure, pulse

1. Administration method of test drug

<SITT> The research subjects will be fasted for at least 12 hours after dinner on the day before the test, and blood vessels will be secured with an indwelling needle for blood collection and insulin administration. Fast-acting insulin at 0.1 unit/kg body weight was diluted to 1 unit/ml with physiological saline in the early morning on an empty stomach and administered as a bolus intravenously over approximately 20 seconds, collect blood (1 ml) before loading, and 3, 6, 9, 12 and 15 minutes after insulin administration, and measure blood glucose level. After blood collection 15 minutes later, 20 ml of 50% glucose solution is administered intravenously to prevent hypoglycemia, and the test is terminated. After intravenous insulin injection, calculate the plasma glucose concentration disappearance rate (Kitt) from the decrease curve of plasma glucose concentration from 3 to 15 minutes.

<Simple insulin tolerance test>

Following the same procedure as the SITT described above, the research subject will be fasted for at least 12 hours after dinner on the day before the test, and a blood vessel for blood sampling will be secured using an indwelling needle. Fast-acting insulin at 0.1 unit/kg body weight was injected subcutaneously in the early morning on an empty stomach, and collect blood(1ml) before loading, 3 , 6, 9, 12, 15, 30, 45 , 60, 75, 90 and 120 minutes after insulin administration, and measure blood glucose. At the same time, a simple blood glucose meter is used to measure blood glucose before loading and at 3, 6, 9, 12, 15, 30, 45, 60, 75, 90, and 120 minutes after insulin administration. Calculate the plasma glucose concentration disappearance rate (Kitt) from the decrease curve of plasma glucose concentration from 15 to 90 minutes.

1. Regulations regarding concomitant drugs (therapy)

Not applicable

1. Regulations regarding dose reduction and drug suspension

Not applicable

1. Expected period of research participation of research subjects

After consenting, each research subject (inpatient) will participate for a one-month observation period. The expected participation period for each research subject (volunteer) is approximately 3 days (2-3 visits to the hospital).

6. Case registration and allocation method

(1) Case registration

The co-researcher shall provide the necessary information to match the consent acquisition date, research subjects, and research subject identification codes in the research subject identification code list kept by the principal investigator. co-researcher will submit a case registration form using the research subject identification code to the research office. The co-researcher will receive confirmation of eligibility from the research office and a registration confirmation letter containing the research subject registration number, etc.

(2) Allocation method

Trial assignments to research subjects will be performed using the central registration method after case registration, and the assignment results will be communicated to the research collaborators from the research office on paper. The specific allocation will be described in the allocation plan.

(3) Blinding

Not applicable

7. Observation and inspection items

Observations and tests will be conducted on the following items, and the data will be used in this research.

- 1. Research subject background (inpatients): initials, identification code, age, gender, diagnosis name, height, weight, BMI, waist circumference, waist-to-hip ratio, disease duration, complications, smoking/drinking, medication intake details

Research subject background (volunteer): initials, identification code, age, gender, height, weight, BMI, waist circumference, waist-hip ratio, presence of smoking/drinking

- 1. Blood pressure, pulse rate
  2. Clinical examination

＜Next day of hospitalization*Early morning fasting blood sampling (inpatients)＞*If the day falls on a holiday, on the weekday after the holiday

Urinalysis (urine glucose, urine protein (qualitative), urine albumin/creatinine ratio), HbA1c, fasting plasma glucose level, insulin, S-C peptide, fasting serum lipids (total cholesterol, triglycerides, HDL cholesterol, LDL cholesterol, RLP-C), AST, ALT, γ-GTP, ChE, uric acid, BUN, serum creatinine, Na, K, Cl, hs-CRP, ACTH, cortisol, hGH, somatomedin, renin, aldosterone, blood catecholamine fraction, Ferritin, hyaluronic acid, type 4 collagen 7S, plasma amino acid concentration

Biomarkers: adiponectin, TNF-α, leptin

Blood collection for storage: There is a possibility that additional items will be measured at a later date, and if consent is obtained from the research subject, blood collection for storage (16ml) will also be performed. Specimens will be stored at Hokkaido University Hospital, Department of Internal Medicine I.

*Blood collection for storage, biomarkers, and plasma amino acid concentrations were conducted for research purposes. Others were conducted for medical purposes.

<Blood collection at the time of visit (volunteer)> *Only once when performing either SITT or simple insulin tolerance test

Fasting plasma glucose level, insulin, S-C peptide, plasma amino acid concentration

Biomarkers: adiponectin, TNF-α, leptin

Blood collection for storage: There is a possibility that additional items will be measured at a later date, and if consent is obtained from the research subject, blood collection for storage (16ml) will also be performed. Specimens will be stored at Hokkaido University Hospital, Department of Internal Medicine I.

<Blood collection during short-term insulin tolerance test (SITT)> Blood glucose (before loading, 3 minutes, 6 minutes, 9 minutes, 12 minutes, 15 minutes after insulin administration)

<Blood collection during simple insulin tolerance test> Blood glucose(before loading, 3 minutes, 6 minutes, 9 minutes, 12 minutes, 15 minutes, 30 minutes, 45 minutes, 60 minutes, 75 minutes, 90 minutes, 120 minutes after insulin administration)

*Underlined part: Blood glucose levels for short-term insulin tolerance tests and simple insulin tolerance tests are outsourced to SRL.

- 1. Body composition analysis: Body composition analysis will be performed using the DXA method and the impedance method (InBody, BIO SPACE), but since it will vary depending on the measurement time and meal intake status, the measurement will be performed in the morning on an empty stomach.
  2. Basal metabolism measurement: Basal metabolism measurement is performed using a basal metabolism measurement device (Metabolic Analyzer, MedGemⓇ), but since it varies depending on the measurement time and meal intake status, the measurement should be performed in the morning on an empty stomach.
  3. Continuous glucose monitoring (CGM) *Performed only on hospitalized patients
  4. CT　*Performed only on hospitalized patients
  5. Simple blood glucose measurement

8. Expected benefits and disadvantages (burdens and risks)

(1) Expected profit

<Research 1> The SITT test conducted in this study allows for accurate assessment of insulin resistance, allowing for the selection of appropriate treatments for diabetic patients. As for volunteers, it is possible to determine whether they have insulin resistance or abnormal glucose tolerance at an early stage, and by giving them appropriate lifestyle guidance, it may be possible to prevent the onset of diabetes. Additionally, the research results may contribute to future medical advances.

<Research 2> The simple insulin tolerance test conducted in this study allows for accurate assessment of insulin resistance, making it possible to select appropriate treatments for diabetic patients. As for volunteers, it is possible to determine whether they have insulin resistance or abnormal glucose tolerance at an early stage, and by giving them appropriate lifestyle guidance, it may be possible to prevent the onset of diabetes. Additionally, the research results may contribute to future medical advances.

(2) Expected disadvantages (side effects)

See attached document for details. The main side effect of Humulin R is reported to be hypoglycemia.

In addition, by participating in Study 1, patients will receive approximately 48 ml of blood (test + blood collection for storage + biomarker + plasma amino acid concentration), and volunteers will receive approximately 52 ml (test + blood collection for storage + biomarker + fasting blood glucose, insulin, S-C peptide, plasma amino acid concentration) is required. When participating in Study 2, patients will have approximately 54 ml of blood collected (test + storage blood collection + biomarker + plasma amino acid concentration), and volunteers will have approximately 58 ml of blood (test + storage blood collection + biomarker + fasting blood glucose, insulin, S-C peptide, plasma amino acid concentration) is required. When conducting a simple insulin tolerance test, 12 simple blood glucose measurements are required in Study 1 and 24 times in Study 2.

In addition, fasting for 12 hours or more is required to perform the SITT or simple insulin tolerance test. SITT takes about 15 minutes, and simple insulin tolerance test takes about 120 minutes.

Volunteers will be required to visit the hospital two to three times (approximately 2 hours per visit).

(3) Comprehensive evaluation of benefits and disadvantages and countermeasures against disadvantages

Monitor blood glucose levels with a simple blood glucose meter, and if hypoglycemia occurs, administer glucose as appropriate. During the SITT test, a simple blood glucose measurement is also performed using the remaining blood from the venous blood draw. If the target patient has hypoglycemia of less than 70 mg/dl during SITT, or if initial symptoms of hypoglycemia (sweating, tremors, palpitations, etc.) are observed even if the blood glucose is 70 mg/dl or more, inject 20 ml 50% glucose solution intravenously. In addition, if the volunteer has hypoglycemia below 60 mg/dl during SITT, or if the volunteer has early symptoms of hypoglycemia (sweating, tremors, palpitations, etc.) even if the blood glucose is over 60 mg/dl, inject 20ml 50% glucose solution orally. Retest the blood glucose level after 15 minutes and repeat the above steps until it is over 100mg/dl. If the target patient has hypoglycemia of less than 70 mg/dl during a simple insulin tolerance test, or if the initial symptoms of hypoglycemia (sweating, tremors, palpitations, etc.) are observed even if the blood glucose is 70 mg/dl or more, take 10 g of glucose internally. In addition, the same applies if a volunteer has hypoglycemia below 60 mg/dl during a simple insulin tolerance test, or if the volunteer exhibits early symptoms of hypoglycemia (sweating, tremors, palpitations, etc.) even if the blood glucose is over 60 mg/dl, Take 10g of glucose orally. Retest the blood glucose level after 15 minutes and repeat the above steps until it is over 100mg/dl.

9. Evaluation item

(1) Primary endpoint

The evaluation of a simple insulin tolerance test is the K index of ITT (Kitt), which is the rate of disappearance of blood glucose from 15 minutes to 90 minutes, and the evaluation of a short insulin tolerance test is the K index of ITT (Kitt) from 3 minutes to 15 minutes, and Lundbaek's calculation. Calculate using formula ⁵⁾ to evaluate the correlation between the results of the simple insulin tolerance test and the short-term insulin tolerance test.

(2) Secondary endpoints

① Measurement of the maximum amount of insulin required to eliminate glucotoxicity ② Frequency of adverse events ③ Analyze the relationship between insulin resistance indicators (HOMA-IR, Kitt) and other clinical indicators such as blood amino acid levels and body composition analysis results.

10. Discontinuation criteria for individual research subjects and responses after research implementation

(1) What to do when research is discontinued

If the person in charge of research determines that it is impossible to continue research on an individual research subject for the following reasons, the research person will discontinue the research on that research subject. In that case, the reason for discontinuation will be explained to the research subjects as necessary. Furthermore, treatment of research subjects after discontinuation will be handled in good faith to ensure that research subjects are not disadvantaged.

(2) Discontinuation criteria

①When a research subject requests to decline research participation or withdraws consent.

1. If it is found that the eligibility is not satisfied after registration
2. If it is difficult to continue the study due to adverse events

④If it is difficult to continue the study due to worsening of complications If it is difficult to continue the study due to adverse events

⑤If the test subject is found to be pregnant

⑥If the entire research is discontinued

1. If the researcher determines that it is appropriate to discontinue the research for other reasons.

(3)Response after research implementation

After conducting this research, the research director will provide the research subjects with the most appropriate medical care, including the results obtained from this research. Regarding volunteers, if we detect any abnormality, we will recommend medical treatment as deemed appropriate.

11. Handling of test results obtained from research on individual research subjects

<Research 1>

The results of SITT will be disclosed to research subjects and reflected in treatment and lifestyle guidance. The results of the simple insulin tolerance test will be disclosed only to those who wish to do so, but explanations on how to interpret the results will not be provided.

<Research 2>

The results of the simple insulin tolerance test will be disclosed and reflected in treatment and lifestyle guidance.

12. Handling when an adverse event occurs

(1) Responses to research subjects when adverse events occur

An adverse event is any undesirable or unintended injury or illness that occurs to a research subject, or its symptoms (including abnormalities in laboratory test values), regardless of whether or not there is a causal relationship with the conducted research. When the researcher observe an adverse event, they will immediately take appropriate measures and record it in the medical records.In addition, the researcher will be informed if administration of the study drug is discontinued or if treatment for an adverse event becomes necessary.

(2) Reporting of serious adverse events

Serious adverse events are defined as follows:

1) Things that lead to death

2) Life-threatening

3) Items that require hospitalization for treatment or an extension of the hospitalization period

4) Things that result in permanent or significant disability or dysfunction

5) Causes birth defects in offspring

If the researcher becomes aware of the occurrence of a serious adverse event, they will take necessary measures such as providing explanations to research subjects, and will promptly report to the research director.

If the research director learns of the occurrence of a serious adverse event while conducting invasive research, the research director shall promptly report the fact to the director of the research institution, take appropriate measures, and promptly report the occurrence of a serious adverse event. Information regarding the occurrence of the adverse event will be shared with the research personnel involved in the implementation of the research.

(3) Reporting of important adverse events

Not applicable

(4) Other adverse events

For other adverse events, the researcher will appropriately record them in the medical records, etc.

13. Approval, changes, and revisions of research implementation plans

The research director shall submit the clinical research plan to the head of the research institution in advance, and obtain approval from the independent clinical research review committee (hereinafter referred to as the review committee) and permission from the head of the research institution regarding the implementation of the research. In addition, when changing or revising the research implementation plan, promptly submit the revised version to the head of the research institution according to the established work procedure, and obtain approval from the review committee and permission from the head of the research institution.

14. Cancellation, interruption, or termination of research

(1) Termination or suspension

The co-researcher will consider whether to continue conducting the research if any of the following apply.

① When they become aware of matters related to safety and efficacy and other important information.

②When it is judged that it is difficult to recruit research subjects and it will be extremely difficult to reach the expected number of cases.

③When the purpose of the research is achieved before the planned number of cases or the planned period is reached.

④When the screening committee has given instructions to change the implementation plan, and it is determined that it is difficult to accept the instructions.

The research director will discontinue the research if the review committee recommends or instructs it to be discontinued. In addition, when a decision is made to discontinue or suspend research, promptly report the reason in writing to the director of the research institution.

(2) Completion of research

Upon completion of the research, the research director will promptly submit a research completion report to the head of the research institution.

15. Research implementation period

Implementation permission date - March 31, 2018

16. Target number of cases, basis for setting it, and statistical analysis method

(1) Target number of cases and basis for setting it

<Research 1> 24 cases (7 volunteers, 17 diabetic patients) [Based for setting] Assuming a correlation coefficient of 0.6, setting a power of 80% and a significance level of 5%, n = 19 cases will be required. The sample was set at n=24, assuming that 20% of cases would drop out.

<Research 2> 11 cases (6 volunteers, 5 diabetic patients) [Based for setting] Assuming that the expected intraclass correlation coefficient for two measurements is 0.8 and the intraclass correlation coefficient for the null hypothesis is 0.1, the required number of subjects is 9 with a significance level of 5% and a power of 80%6⁾. The sample was set at n=11, assuming that 20% of cases would drop out.

(2) Statistical analysis method

For study 1, Pearson's correlation coefficient was calculated for the correlation between Kitt (SITT) calculated from the SITT results and Kitt (i.m.) calculated from the simple insulin tolerance test, and a correlation coefficient of 0 was set as the null hypothesis. Perform a two-tailed test. For estimation of the calibration straight line, orthogonal regression with a variance ratio of 1 is performed.

For Study 2, Fisher's Z transformation was performed on the intraclass correlation coefficient, and the null hypothesis was tested at 2.5% on one side6⁾. Other statistical analyzes will be described in the statistical analysis plan.

17. Consideration for the human rights of research subjects

All researchers in this research must comply with the Declaration of Helsinki (revised in October 2013) and the Ethical Guidelines for Medical Research Involving Human Subjects (Ministry of Education, Culture, Sports, Science and Technology/Ministry of Health, Labor and Welfare Notification No. 3 of 2014).

18. Handling of personal information

When handling samples and information related to research implementation, sufficient consideration should be given to protecting the confidentiality of research subjects by assigning numbers unrelated to the personal information of the research subjects and managing them in a linkable and anonymized manner. The research director will supervise that the anonymization correspondence table is stored in strict confidence. In addition, when research directors and others publish information obtained through this research, they will ensure that it does not include information that could identify research subjects.

19. How to obtain consent

The person in charge of the research will give the consent explanation document approved by the review committee to the research subject, provide sufficient written and verbal explanations, and obtain the free voluntary consent of the research subject in writing. When information that affects the consent of research subjects is obtained, or when changes are made to the implementation plan that may affect the consent of research subjects, the person in charge of the research shall promptly notify the research subjects. In addition to providing information and confirming the research subject's intention in advance as to whether or not to participate in the research, we will revise the consent explanation document, etc. with prior approval from the review committee, and obtain re-consent from the research subject.

1. The name of the research and the fact that permission has been obtained from the head of the research institution to conduct the research.
2. Name of research institution and name of research director
3. Purpose and significance of the research
4. Research method (including purpose of use of samples and information obtained from research subjects) and period
5. Reason for being selected as a research subject
6. Burdens incurred by research subjects and anticipated risks and benefits
7. Even if you agree to the conduct or continuation of the research, you may withdraw your consent at any time (if it becomes difficult to take measures in accordance with the details of the withdrawal from the research subjects, etc., That fact and the reason)
8. A statement that research subjects will not be treated disadvantageously by not consenting to the conduct or continuation of the research or by withdrawing consent.
9. Method of disclosing information regarding research
10. At the request of research subjects, etc., materials related to research plans and research methods can be obtained or viewed to the extent that it does not interfere with the protection of personal information of other research subjects and ensuring the originality of the research concerned.
11. Handling of personal information(including the method of anonymization)
12. Methods for storing and disposing of samples and information
13. Conflicts of interest related to research by research institutions, such as funding sources for research, and conflicts of interest related to research by researchers, such as personal earnings.
14. Responding to consultations, etc. from research subjects and their related parties
15. If there is a financial burden or reward on the research subjects, etc., that fact and its contents (this research does not apply)
16. In the case of research that involves medical actions beyond normal medical treatment, matters related to other treatment methods, etc. (this research does not apply)
17. In the case of research that involves medical actions beyond normal medical treatment, responses regarding the provision of medical care to research subjects after the research is conducted (this research does not apply)
18. In cases where there is a possibility that important knowledge regarding the health of the research subject or genetic characteristics that may be passed on to offspring may be obtained as a result of the research, the results of the research concerning the research subject (including incidental findings may be obtained).
19. In the case of invasive research, the presence or absence of compensation for health damage caused by the research and its details.
20. If there is a possibility that samples and information obtained from research subjects may be used for future research that is not specified at the time of obtaining consent from the research subjects, or may be provided to other research institutions, Contents expected at the time of receiving consent to that effect

㉑A statement that those engaged in monitoring and the review committee will view samples and information regarding the research subject to the extent necessary, on the premise that the confidentiality of the research subject will be maintained.

20. Response to and compensation for health damage to research subjects

(If the research subjects are patients) If any health damage occurs to the research subjects as a result of conducting this research, the person in charge of the research will take appropriate measures. Compensation for health damage will be provided in accordance with the "Ethical Guidelines for Medical Research Involving Human Subjects." In other words, compensation will be provided for death or residual disability (class 1 and class 2 disability) resulting from this research. For any other health damage, necessary measures such as testing and treatment will be provided under the health insurance of the research subjects.

(If the research subjects are healthy individuals) If any health damage occurs to the research subjects as a result of conducting this research, the person in charge of the research will take appropriate measures. Compensation for health damage will be provided in accordance with the "Ethical Guidelines for Medical Research Involving Human Subjects." In other words, compensation will be provided for death or residual disability (disability grades 1 to 14) that occur as a result of this research. For any other health damage, necessary measures such as testing and treatment will be provided under the health insurance of the research subjects.

21. Contents and method of reporting to the head of the research institution.

(1) Progress report

The research director shall report the progress of the research and the occurrence of adverse events associated with the implementation of the research in writing to the head of the research institution at least once a year.

(2) Reporting of serious adverse events

If the research director becomes aware of the occurrence of a serious adverse event, he/she shall promptly report the fact to the director of the research institution.

(3) When information such as facts that impair the ethical validity or scientific rationality of the research is obtained.

If the research director obtains any facts or information that impair the ethical validity or scientific rationality of the research, or any information that is likely to impair the research and is considered to affect the continuation of the research, the research director shall do so without delay. Report this to the head of the research institution.

(4) If we obtain information such as facts that undermine the appropriateness of conducting research or the credibility of research results.

If the person in charge of research obtains facts or information that impair the appropriateness of the conduct of the research or credibility of the research results, or information that may impair the credibility, the researchers in this research shall promptly report the fact to the director of the research institution.

(5) Reporting of research completion (including in the case of cancellation)

When the research director has completed the research, he shall report the fact and a summary of the research results in writing to the director of the research institution without delay in accordance with 14.

(6) Management status of samples and information used in research

The research director shall manage the storage of samples and information obtained from human bodies as necessary in accordance with 24.(1)(2), and shall report the management status to the head of the research institution.

(7)Report on publication of research results

When the research director has made the final publication of the results, he shall report to the head of the research institution without delay in accordance with 25. In addition, if the research results are to be published after the final publication has been reported, promptly report this to the head of the research institution.

22. Efficacy and Safety Evaluation Committee

Not applicable

23. Cost burden for research subjects

The research is responsible for the administration of drugs used in this study and the tests performed (fasting plasma glucose level, insulin, S-C peptide, plasma amino acid concentration, adiponectin, TNF-α, leptin, SITT blood glucose level, and simple insulin tolerance test blood glucose level). This will be covered by research funds from the medical department to which the person belongs. Other than that, research subjects will not have to pay any costs for participating in the study, as it will be covered under normal medical insurance. As for volunteers, research subjects will not be burdened with any costs associated with participating in the research.

Please note that no compensation will be provided to research subjects.

24. How to store and dispose of samples and information

(1) Methods for storing and disposing of samples

The research director will instruct the research personnel to properly store the specimens according to established storage methods, and will carry out necessary controls to prevent leakage, mixing, theft, and loss of specimens.

The collected blood (residue after measurement, blood collected for storage) will be frozen and stored at the Internal Medicine Department until 5 years after the study ends, provided the consent of the research subjects is obtained. If the consent of the research subject cannot be obtained, the device will be discarded immediately after measurement.

When disposing of it, anonymize it and be careful about personal information.

(2) Information storage and disposal methods

The research director will instruct research personnel to ensure that information (information used in research and materials related to the information) is accurate according to established storage methods, and to prevent information leakage, mixing, theft, loss, etc. Perform necessary management to prevent this from happening.

The information obtained in this study will be stored in a locked cabinet in the Internal Medicine Ward I of the medical office.

The research director shall retain the information used in the research for as long as possible, and shall report at least five years after the date on which the completion of the research is reported or the final publication of the results of the research. Store the information appropriately until the later of three years from the date on which it was made. Additionally, if the hospital has a correspondence table for linkable and anonymized information, the same shall apply to the storage of the correspondence table.

When disposing of it, anonymize it and be careful about personal information.

(3) Regarding secondary use of samples and information

Samples and information of research subjects obtained in this study may be used for future research that will not be specified at the time of consent. In that case, the research will be conducted after providing a separate explanation to the research subjects.

25. Methods of disclosing information regarding research and publication of research results

The research director will register the outline of the research in the public database prior to its implementation, and will update it as appropriate according to changes in the research plan and the progress of the research. When research is completed, the results of the research shall be registered without delay. Furthermore, when making public the results, necessary measures will be taken to protect the human rights of research subjects and their associates, or the rights and interests of research personnel and their associates.

When final results are published, report to the head of the research institution without delay. The public database to be registered will be the University Hospital Medical Information Network (UMIN-CTR).

26. Regarding ownership of intellectual property rights

If results are obtained from this research and intellectual property rights arise, those rights belong to our research group.

27. Research funding and conflicts of interest

This research will be conducted with research funds from the medical department to which the principal investigator belongs. In addition, the researcher in this study shall declare the necessary matters to the Conflict of Interest Review Committee and obtain its review and approval in accordance with the provisions of the "Internal Regulations for Conflict of Interest Management for Clinical Research at Hokkaido University Hospital."

28. Monitoring

The research director will strive to ensure the reliability of the research, and ensure that the human rights, safety, and welfare of the research subjects are protected, that the research is conducted in compliance with the research implementation plan, and that the research director Monitoring is conducted to ensure that data reported by individuals is being accurately collected. The research director shall designate a person in charge of monitoring to ensure that monitoring is carried out appropriately. The person in charge of monitoring will carry out monitoring according to the monitoring procedure manual prepared in advance.

29. Audit

In this research, quality control will be properly implemented through monitoring and no auditing will be conducted.

30. Research implementation system

This research will be conducted under the following structure.

[Research Director]

Taku Watanabe (Assistant Professor, Department of Internal Medicine I, Hokkaido University Hospital)

011-706-5911 (extension 35911)

*For research collaborators, please refer to Form A-2 “List of independent clinical research collaborators”

[contact address]

Internal Medicine I Medical Office 011-706-5911 (extension 35911)

Inpatient Ward 011-706-5808 (extension 35808)

Outpatient department 011-706-5752 (extension 35752)

[Consultation counter]

Internal Medicine I Inpatient Ward 011-706-5808 (Extension 35808)

[Data management facility]

Akiko Hayashishita (Hokkaido University Hospital Internal Medicine I)

E-mail: [akiko.hayashishita@pop.med.hokudai.ac.jp](mailto:akiko.hayashishita@pop.med.hokudai.ac.jp)

PHS82377

[Research Office]

Hokkaido University Internal Medicine I Contact information: Kita 15-jo Nishi 7-chome, Kita-ku, Sapporo, Hokkaido 060-0838

Hokkaido University Hospital Internal Medicine I Medical Department

011-706-5911

[Statistical analysis manager]

Yoichi Ito (Hokkaido University Graduate School of Medicine, Department of Social Medicine)

[Monitoring facilities]

Masaru Suzuki ( Department of Internal Medicine I, Hokkaido University Hospital)

011-706-5911 (extension 35911)

[Registration Office]

Exum Co., Ltd.

〒060-0001 2 Kita 1-jo Nishi 5-chome, Chuo-ku, Sapporo

TEL: 011-222-5225 FAX: 011-222-5265

[Assignment person] Keigo Nakajima (Exum Co., Ltd.)

011-222-5225

[External support organization]

SRL Co., Ltd.

Hokkaido Laboratory Address: 2-25 Minami 19-jo Nishi 13-chome, Chuo-ku, Sapporo 064-0919

TEL: 011-511-9991 FAX: 011-520-8161

Based on the outsourcing contract, samples will be collected from research participating facilities and measured.

31. Reference materials/literature list

1) Diabetes specialist training guidebook, revised 6th edition. Edited by the Japan Diabetes Society. Diagnosis and Treatment Co., Ltd.

2)Matthews DR et al: Diabetologia. 1985; 28: 412-419

3)Rabinowitz D et al: J Clin Invest. 1962; 41: 2173-2181

4)Bonora E et al: J Clin Endocrinol Metab. 1989; 68: 374-378

5)Lundbaek K et al: British Medical Journal. 1962; J 2: 1507-1513

6)Machin D et al: Sample size tables for clinical studies. 3^rd^ ed. 2009. Wiley-Blackwell.
