# Supplementary material for: Insulin resistance assessed by short insulin tolerance test and its association with obesity and insulin resistance-related parameters in humans: A pilot randomized trial
Source: PLoS One. 2024 Jun 21;19(6):e0297718. doi: 10.1371/journal.pone.0297718 (PMC11192359; doi:10.1371/journal.pone.0297718)
Supplement: S2 Protocol — (DOCX) [file pone.0297718.s003.docx]

自主臨床研究

**糖尿病患者における簡易インスリン負荷試験を**

**用いたインスリン抵抗性評価法の検討**

**研　究　実　施　計　画　書**

研究代表者 北海道大学病院 内科Ⅰ 渡部　拓

作成日

2016年3月29日　計画書　第1版作成

2016年5月30日　計画書　第1.1版作成

2016年7月 1日　計画書　第1.2版作成

2016年8月24日　計画書　第1.3版作成

2016年9月12日　計画書　第1.4版作成

**目　次**

[**1.** **研究の背景** 3](#_Toc457239399)

[**2.** **研究の目的** 3](#_Toc457239400)

[**3.** **試験薬の概要** 3](#_Toc457239401)

[**4. 対象者および適格性の基準** 4](#_Toc457239402)

[**5.** **研究の方法** 5](#_Toc457239401)

[**6．症例登録、割付方法** 8](#_Toc457239403)

[**7.** **観察および検査項目** 8](#_Toc457239404)

[**8．予想される利益および不利益（負担およびリスク）** 10](#_Toc457239405)

[**9．評価項目** 11](#_Toc457239406)

[**10．個々の研究対象者における中止基準および研究実施後の対応** 11](#_Toc457239407)

[**11．個々の研究対象者における研究によって得られた検査結果の取扱い** 11](#_Toc457239408)

[**12．有害事象発生時の取扱い** 12](#_Toc457239409)

[**13．研究実施計画書等の承認・変更、改訂** 12](#_Toc457239410)

[**14．研究の中止・中断、終了** 12](#_Toc457239411)

[**15．研究実施期間** 13](#_Toc457239412)

[**16．目標症例数とその設定根拠及び統計解析方法** 13](#_Toc457239413)

[**17．研究対象者の人権に対する配慮** 13](#_Toc457239414)

[**18．個人情報の取扱い** 13](#_Toc457239415)

[**19．同意取得方法** 14](#_Toc457239416)

[**20．研究対象者の健康被害への対応と補償** 15](#_Toc457239417)

[**21．研究機関の長への報告内容及び方法** 15](#_Toc457239418)

[**22．効果・安全性評価委員会** 16](#_Toc457239419)

[**23．研究対象者の費用負担** 16](#_Toc457239420)

[**24．試料・情報等の保管及び廃棄の方法** 16](#_Toc457239421)

[**25．研究に関する情報公開の方法及び研究結果の公表** 17](#_Toc457239422)

[**27．研究資金及び利益相反** 17](#_Toc457239423)

[**28．モニタリング** 17](#_Toc457239424)

[**29．監査** 17](#_Toc457239426)

[**30．研究実施体制** 17](#_Toc457239428)

[**31．参考資料・文献リスト** 18](#_Toc457239429)

1. **研究の背景**

糖尿病は膵β細胞からのインスリン分泌の低下と標的臓器でのインスリン抵抗性が複雑にからみあって発症する疾患であり、その病態の把握には両者を正確に評価することが重要である。インスリン抵抗性を評価する方法の中で、グルコースクランプ法¹⁾やミニマルモデル法¹⁾は精密であるが手法が煩雑で被験者に対する負担も大きく日常診療でルーチンで行うのは困難である。一方、HOMA法は空腹時の血糖値と血中インスリン値のみから算出できる簡便性から日常診療でしばしば用いられているが、インスリン分泌低下が強くなる空腹時血糖140mg/dl以上の患者やインスリン治療中の患者においては指標として不正確となる短所もある²⁾。また、比較的簡便なインスリン抵抗性評価法として1962年にRabinowitzとZierler³⁾によりインスリン負荷試験が報告されたが、被験者に低血糖症状が出現することが多いことや、急激な血糖低下により、グルカゴン、カテコラミン、GH、コルチゾールなどのインスリン拮抗ホルモンの反応性分泌が生じ結果に影響を与えてしまうことから最近では使用されなくなっている。その後、通常のインスリン負荷試験による低血糖などの欠点を改善した短時間インスリン負荷試験(SITT)がBonoraら⁴⁾により提案され、臨床の現場や臨床論文にもインスリン抵抗性の指標として用いられるようになっている。

我々は、治療内容や血糖値の影響を受けずに正確にインスリン抵抗性を評価できるインスリン負荷試験・SITT法の利点を活かし、さらに簡便で、侵襲が少なく、低血糖のリスクが低いインスリン皮下注射によりインスリン抵抗性を評価するインスリン負荷試験（以下、簡易インスリン負荷試験）を考案した。本試験では簡易インスリン負荷試験によりインスリン抵抗性の評価が可能かどうかをSITT法と比較し有用性を検討する。さらに、SITT法、簡易法で評価した結果をもとに、インスリン抵抗性とインスリン最大使用量との関連についても検討する（研究１）。

また、研究１をすべて終了後、簡易インスリン負荷試験の有用性を評価したのちに、本試験の再現性に関しても評価する（研究２）。

1. **研究の目的**

当科に教育入院中の糖尿病患者に対し、簡易インスリン負荷試験、SITT法を施行し、簡易インスリン負荷試験によりインスリン抵抗性の評価が可能かどうかの検討を行う。さらに、良好な血糖コントロールを得るまでに要した最大インスリン使用量とインスリン抵抗性との関連についても検討する。また、耐糖能異常の有無によるインスリン抵抗性の層別化のため、過去に糖尿病を指摘されたことがないボランティアに対しても簡易インスリン負荷試験、SITT法を施行し比較する。

1. **試験薬の概要**

ヒューマリンⓇＲ注

・一般名：インスリン　ヒト（遺伝子組み換え）注射液

・処方箋医薬品、薬価収載(2008年12月)

・製造・販売元：日本イーライリリー株式会社

・作用機序：肝臓、骨格筋および脂肪に発現しているインスリン受容体に結合し血糖降下作用を発揮する

・適応症：インスリン療法が適応となる糖尿病

・用法・用量：通常、成人では初期は1回4～20単位を一般に毎食前に皮下注射するが、ときに回数を増やしたり、他のインスリン製剤を併用する。以降症状及び検査所見に応じて投与量を増減するが、維持量は通常成人1日4～100単位である。ただし、必要により上記用量を超えて使用することがある。糖尿病昏睡には、必要に応じ皮下、筋肉内、静脈内注射又は持続静脈内注入を行う。

・有害事象：承認時における安全性評価対象例1428例中27例(1.89％)に副作用が報告され、主なものは注射部位障害13例（0.91%）、AST/ALT上昇4例(0.28%)。使用成績調査における安全性評価対象例4588例中494例（10.77%）に臨床検査値の異常変動を含む副作用が報告され、このうち主なものは低血糖等の代謝・栄養障害455例(9.70%)。

・臨床使用成績：インスリン投与を必要とする糖尿病患者を対象として行われた臨床試験の成績の概要（n=124）。平均HbA1c：1～2か月：-0.09%、3～4か月:+0.05%、5～6か月：+0.21%。※HbA1c値はJDS値/NGSP値として標準化される前に測定された値である。

・薬価： 330円/ml

＊詳細は添付文書を参照のこと。

＊＊健康成人に本剤を0.1単位/kg皮下注した際の血糖値の推移は、添付文書【薬物動態】の項を参照。

**4. 対象者および適格性の基準**

（1）対象者のうち、（2）選択基準をすべて満たし、かつ（3）除外基準のいずれにも該当しない場合を適格とする。

（1）対象者

北海道大学病院内科Ⅰに入院中の糖尿病患者、および過去に糖尿病や低血糖を呈する疾患を指摘されていないボランティア。インスリン抵抗性の評価方法としての有用性を検討する研究のため、インスリン抵抗性の程度の異なる、糖尿病患者・ボランティアを対象とする。

（2）選択基準（入院患者）

①同意取得時において年齢が20歳以上の者

②入院翌日（休日・祝日の場合は休み明けの平日）の採血の早朝空腹時血糖が140mg/dl以上の糖尿病患者

③本研究への参加にあたり十分な説明を受けた後、十分な理解の上、本人の自由意思による文書同意が得られた患者

選択基準（ボランティア）

1. 同意取得時において年齢が30歳以上、およびBMI 18.5kg/㎡以上の者
2. 過去に糖尿病や低血糖を呈する疾患を指摘されていない者
3. 本研究への参加にあたり十分な説明を受けた後、十分な理解の上、本人の自由意思による文書同意が得られた者

（3）除外基準（入院患者）

① 糖尿病網膜症の状態が安定していない者

1. 糖尿病腎症4期以上の者
2. 未治療の虚血性心疾患を合併している者
3. てんかん患者
4. 本試験で使用する薬剤の成分に対し、過敏症の既往のある者
5. 妊婦、授乳婦または妊娠している可能性のある者
6. その他、研究責任者または研究分担者が研究対象者として不適当と判断した者

除外基準（ボランティア）

1. 過去に低血糖（血糖60mg/dl未満）を呈する疾患を指摘されている者
2. 本試験で使用する薬剤の成分に対し、過敏症の既往のある者
3. 妊婦、授乳婦または妊娠している可能性のある者
4. 30歳未満、およびBMI 18.5kg/㎡未満の者
5. 未治療の虚血性心疾患を合併している者
6. てんかん患者
7. その他、研究責任者または研究分担者が研究対象者として不適当と判断した者

（4）ボランティアの募集方法

別紙の募集用資料を北海道大学内に掲示し、ボランティアを募る。

1. **研究の方法**

（1）研究の種類・デザイン

検証的臨床試験

（2）試験のアウトライン

　　研究１をすべて終了したのち、研究１で得られた臨床データを統計解析し、簡易インスリン負荷試験の有用性が示唆された場合は、簡易インスリン負荷試験の再現性を検証するため研究２を行う。入院患者は研究１・研究２いずれかの一方の研究に参加する。ボランティアに関しては、両方の研究に参加可能とする。

＜研究１＞

1. 北大病院内科Ⅰに入院中の糖尿病患者のうち、食事療法（25～30Kcal/標準体重）

にて、入院翌日（休日・祝日の場合は休み明けの平日）の早朝空腹時採血で血糖140mg/dl以上の患者を対象に、SITTと簡易インスリン負荷試験を各々別の日(3日以内)に行う。血漿ぶどう糖濃度の減少曲線より血漿ぶどう糖濃度消失率（Kitt）を算出する。Kitt値は値が小さい方がインスリン抵抗性が強いことを表す。

さらに、良好な血糖コントロール（早朝空腹時血糖130mg/dl未満、食後2時間血糖値180mg/dl未満）を達成できるまでに要した最大インスリン必要量の測定、インピーダンス法による体組成・基礎代謝測定、CTによる臍高部での体脂肪面積測定・筋肉量測定、非アルコール性脂肪性肝疾患の評価、ウエストヒップ比の計測を行い、簡易インスリン負荷試験の結果とあわせて、インスリン抵抗性評価法としての有用性を検討する。

糖毒性解除に要したインスリンの総量

SITTあるいは簡易インスリン負荷試験

SITTあるいは簡易インスリン負荷試験

同意取得

適格性の確認

CT、体組成・基礎代謝測定（入院期間中に行う）

患者背景、血圧、脈拍、採血、尿検査

CGM開始

プラセボ1回1錠1日1回朝食後服用

入院

(2週間)

両検査は3日以内に行う。

SITTと簡易インスリン負荷試験の実施順はランダムに割り付ける。

入院

(2週間)

(12週間)

入院期間

(約1か月)

1. ボランティアを対象に、SITTと簡易インスリン負荷試験を各々別の日（3日以内）に行う。血漿ぶどう糖濃度の減少曲線より血漿ぶどう糖濃度消失率（Kitt）を算出する。Kitt値は値が小さい方がインスリン抵抗性が強いことを表す。

さらに、インピーダンス法による体組成・基礎代謝測定、ウエストヒップ比の計測を行い、簡易インスリン負荷試験の結果とあわせて、インスリン抵抗性評価法としての有用性を検討する。

同意取得

SITTあるいは簡易インスリン負荷試験

SITTあるいは簡易インスリン負荷試験

適格性の確認

採血、体組成・基礎代謝測定

患者背景、血圧、脈拍

プラセボ1回1錠1日1回朝食後服用

入院

(2週間)

両検査は3日以内に行う。

SITTと簡易インスリン負荷試験の実施順はランダムに割り付ける。

入院

(2週間)

(12週間)

＜研究２＞

- 1. 北大病院内科Ⅰに入院中の糖尿病患者のうち、食事療法（25～30Kcal/標準体重）にて、入院翌日（休日・祝日の場合は休み明けの平日）の早朝空腹時採血で血糖140mg/dl以上の患者を対象に、簡易インスリン負荷試験を別の日に2回行い（3日以内）、簡易インスリン負荷試験の再現性を確認する。

簡易インスリン負荷試験②

簡易インスリン負荷試験①

同意取得

適格性の確認

体組成・基礎代謝測定、CT（入院期間中に行う）

患者背景、血圧、脈拍、採血、尿検査

プラセボ1回1錠1日1回朝食後服用

入院

(2週間)

CGM開始

入院期間

(約1か月)

両検査は３日以内に行う。

- 1. ボランティアを対象に簡易インスリン負荷試験を別の日に2回行い（3日以内）、簡易インスリン負荷試験の再現性を確認する。

簡易インスリン負荷試験②

簡易インスリン負荷試験①

同意取得

適格性の確認

両検査は3日以内に行う

採血、体組成・基礎代謝測定

患者背景、血圧、脈拍

（3）試験薬の投与方法

＜SITT＞

研究対象者を試験前日夕食後から12時間以上絶食とし、留置針で採血及びインスリン投与のための血管を確保する。早朝空腹時に0.1単位/kg 体重の速効型インスリンを生理食塩水で1単位/mlに希釈し約20秒かけて静脈内へbolus投与し、負荷前、インスリン投与後3分、6分、9分、12分、15分に採血（1ml）して血糖値を測定する。15分後の採血後に低血糖を予防するために50%ぶどう糖液20mlを静脈内投与し、試験を終了する。インスリン静注後、3-15分の血漿ぶどう糖濃度の減少曲線より血漿ぶどう糖濃度消失率（Kitt）を算出する。

＜簡易インスリン負荷試験＞

上記SITTと同様の手順で、研究対象者を試験前日夕食後から12時間以上絶食とし、留置針で採血のための血管を確保する。早朝空腹時に0.1単位/kg体重の速効型インスリンを皮下注射し、負荷前、インスリン投与後3分、6分、9分、12分、15分、30分、45分、60分、75分、90分、120分に採血(1ml)して血糖を測定する。同時に簡易血糖測定器でも負荷前、インスリン投与後3分、6分、9分、12分、15分、30分、45分、60分、75分、90分、120分に血糖測定を行う。15-90分までの血漿ぶどう糖濃度の減少曲線より血漿ぶどう糖濃度消失率（Kitt）を算出する。

（4）併用薬（療法）についての規定

該当なし

（5）減量及び休薬についての規定

　該当なし

（6）研究対象者の研究参加予定期間

各研究対象者（入院患者）は同意後、1ヶ月の観察期間で参加する。

各研究対象者（ボランティア）の参加予定期間は3日程度（来院は2～3回）。

**6．症例登録、割付方法**

（1）症例登録

研究責任者又は研究分担者（以下、研究担当者）は、研究責任者が保管する研究対象者識別コードリストに、同意取得日及び研究対象者と研究対象者識別コードを対応させるために必要な事項を記載する。研究担当者は、研究対象者識別コードを用いた症例登録書を、研究事務局に提出する。研究担当者は、研究事務局から適格性の確認を受け、研究対象者登録番号等が記載された登録確認書を受領する。

（2）割付方法

研究対象者への試験の割付は、症例登録後、中央登録法で割付を行い、割付結果は紙面で研究事務局から研究分担者に伝えられる。具体的な割付については割付計画書に記載される。

（3）盲検化

該当なし

1. **観察および検査項目**

以下の項目について、観察および検査を実施し、そのデータを本研究に利用する。

1. 研究対象者背景（入院患者）：イニシャル、識別コード、年齢、性別、診断名、身長、体重、BMI、腹囲、ウエストヒップ比、罹病期間、合併症、喫煙・飲酒の有無、薬剤服薬内容

研究対象者背景（ボランティア）：イニシャル、識別コード、年齢、性別、身長、体重、BMI、腹囲、ウエストヒップ比、喫煙・飲酒の有無

1. 血圧、脈拍数
2. 臨床検査

＜入院翌日*早朝空腹時採血（入院患者）＞*休日・祝日の場合は休み明けの平日

尿検査（尿糖、尿蛋白(定性)、尿中アルブミン/クレアチニン比）、HbA1c、空腹時血糖値、インスリン、S-Cペプチド、空腹時血清脂質(総コレステロール、中性脂肪、HDLコレステロール、LDLコレステロール、RLP-C)、AST、ALT、γ-GTP、ChE、尿酸、BUN、血清クレアチニン、Na、K、Cl、hs-CRP、ACTH、コルチゾール、hGH、ソマトメジン、レニン、アルドステロン、血中カテコラミン分画、フェリチン、ヒアルロン酸、4型コラーゲン7S、血漿アミノ酸濃度

バイオマーカー：アディポネクチン、TNF-α、レプチン

保管用採血：後日、追加項目の測定をする可能性があり、研究対象者の同意が得られた場合、保管用採血（16ml）も行う。検体の保管は北海道大学病院内科Ⅰにて行う。

※保存用採血、バイオマーカー、血漿アミノ酸濃度は、研究目的で実施。それ以外は診療目的で実施。

＜来院時*採血（ボランティア）＞*SITT、簡易インスリン負荷試験いずれか施行時に１回のみ

空腹時血糖値、インスリン、S-Cペプチド、血漿アミノ酸濃度

バイオマーカー：アディポネクチン、TNF-α、レプチン

保管用採血：後日、追加項目の測定をする可能性があり、研究対象者の同意が得られた場合、保管用採血（16ml）も行う。検体の保管は北海道大学病院内科Ⅰにて行う。

＜短時間インスリン負荷試験(SITT)時採血＞

血糖（負荷前、インスリン投与後3分、6分、9分、12分、15分）

＜簡易インスリン負荷試験時採血＞

血糖（負荷前、インスリン投与後3分、6分、9分、12分、15分、30分、45分、60分、75分、90分、120分）

＊下線部分、短時間インスリン負荷試験・簡易インスリン負荷試験の血糖値はSRLに委託。

1. 体組成分析：体組成分析はDXA法と体組成分析装置（InBody, BIO SPACE）を用いて行うが、測定時間、食事摂取状況により変動するため、測定は空腹で午前中の施行とする。
2. 基礎代謝測定：基礎代謝測定は基礎代謝測定装置（メタボリックアナライザー，MedGem）を用いて行うが、測定時間、食事摂取状況により変動するため、測定は空腹で午前中の施行とする。
3. Continuous glucose monitoring(CGM)※入院患者のみ施行
4. CT　※入院患者のみ施行
5. 簡易血糖測定

**8．予想される利益および不利益（負担およびリスク）**

（1）予想される利益

＜研究1＞

本研究で実施するSITT検査にて、インスリン抵抗性の正確な評価ができ、糖尿病患者に対し適切な治療の選択が可能となる。ボランティアに関しては、インスリン抵抗性の有無・耐糖能異常の有無を早期に把握することが可能であり、適切な生活指導を行うことにより糖尿病発症の予防につながる可能性がある。また、研究成果により将来の医療の進歩に貢献できる可能性がある。

＜研究2＞

本研究で実施する簡易インスリン負荷試験にて、インスリン抵抗性の正確な評価ができ、糖尿病患者に対し適切な治療の選択が可能となる。ボランティアに関しては、インスリン抵抗性の有無・耐糖能異常の有無を早期に把握することが可能であり、適切な生活指導を行うことにより糖尿病発症の予防につながる可能性がある。また、研究成果により将来の医療の進歩に貢献できる可能性がある。

（2）予想される不利益（副作用）

詳細は添付文書を参照。ヒューマリンRの主な副作用は低血糖が報告されている。また研究１に参加することにより患者は48ml程度の採血（試験+保管用採血+バイオマーカー+血漿アミノ酸濃度）、ボランティアは52ml程度（試験＋保管用採血＋バイオマーカー＋空腹時血糖、インスリン、S-Cペプチド、血漿アミノ酸濃度）の採血が必要となる。研究2に参加する場合は、患者は54ml程度の採血（試験+保管用採血+バイオマーカー+血漿アミノ酸濃度）、ボランティアは58ml程度（試験＋保管用採血＋バイオマーカー＋空腹時血糖、インスリン、S-Cペプチド、血漿アミノ酸濃度）の採血が必要となる。

簡易インスリン負荷試験実施の際は、研究１では12回、研究2では24回の簡易血糖測定が必要になる。

また、SITT、簡易インスリン負荷試験を実施するにあたり、12時間以上の絶食が必要となる。SITTは約15分、簡易インスリン負荷試験は約120分の検査時間を要する。

ボランティアに関しては、2～3回の来院（1回の来院につき2時間程度）が必要となる。

（3）利益及び不利益の総合的評価と不利益に対する対策

簡易血糖測定器で血糖値をモニタリングし、低血糖時は、適宜、ぶどう糖投与で対応する。SITT検査中は、静脈採血の残血で簡易血糖測定も行う

SITT中に対象患者に70mg/dl未満の低血糖を認めた場合、あるいは血糖70mg/dl以上でも低血糖の初期症状（発汗・震戦・動悸など）を認めた場合、50%ぶどう糖液20mlを静脈注射する。また、SITT中にボランティアに60mg/dl未満の低血糖を認めた場合、あるいは血糖60mg/dl以上でも低血糖の初期症状（発汗・震戦・動悸など）を認めた場合も同様に50%ぶどう糖液20mlを静脈注射する。15分後に血糖値を再検し100mg/dl以上になるまで上記対応を繰り返す。

簡易インスリン負荷試験中に対象患者に70mg/dl未満の低血糖を認めた場合、あるいは血糖70mg/dl以上でも低血糖の初期症状（発汗・震戦・動悸など）を認めた場合、ぶどう糖を10g内服する。また、簡易インスリン負荷試験中にボランティアに60mg/dl未満の低血糖を認めた場合、あるいは血糖60mg/dl以上でも低血糖の初期症状（発汗・震戦・動悸など）を認めた場合も同様にぶどう糖を10g内服する。15分後に血糖値を再検し100mg/dl以上になるまで上記対応を繰り返す。

**9．評価項目**

（1）主要評価項目

簡易インスリン負荷試験の評価は15分-90分までの血糖の消失率であるK index of ITT(Kitt)を、短時間インスリン負荷試験の評価は3分-15分までのKittを、Lundbaekの計算式⁵⁾を用いて算出し、簡易インスリン負荷試験と短時間インスリン負荷試験の結果の相関を評価する。

1. 副次的評価項目
   1. 糖毒性解除に要した最大インスリン必要量の測定
   2. 有害事象発生頻度
   3. インスリン抵抗性の指標（HOMA-IR、Kitt）と 血中アミノ酸値・体組成分析結果などの他の臨床指標との関連を解析する。

**10．個々の研究対象者における中止基準および研究実施後の対応**

（1）研究中止時の対応

研究担当者は、次に挙げる理由で個々の研究対象者について研究継続が不可能と判断した場合には、当該研究対象者についての研究を中止する。その際は、必要に応じて中止の理由を研究対象者に説明する。また、中止後の研究対象者の治療については、研究対象者の不利益とならないよう、誠意を持って対応する。

1. 中止基準
   - 1. 研究対象者から研究参加の辞退の申し出や同意の撤回があった場合
     2. 登録後に適格性を満足しないことが判明した場合
     3. 有害事象により試験の継続が困難な場合
     4. 合併症の増悪により試験の継続が困難な場合
     5. 妊娠が判明した場合
     6. 本研究全体が中止された場合
     7. その他の理由により、研究担当者が研究の中止が適当と判断した場合

（3）研究実施後の対応

本研究実施後は、この研究で得られた成果も含めて、研究責任者は研究対象者に対し最も適切と考える医療を提供する。ボランティアに関しては、異常を認めた際は、適切と考える医療を勧める。

**11．個々の研究対象者における研究によって得られた検査結果の取扱い**

＜研究1＞

SITTの結果を研究対象者に開示し、治療・生活指導に反映する。簡易インスリン負荷試験の結果に関しては希望者のみ開示するが、結果の解釈についての説明は行わない。

＜研究2＞

簡易インスリン負荷試験の結果を開示し、治療・生活指導に反映する。

**12．有害事象発生時の取扱い**

（1）有害事象発生時の研究対象者への対応

有害事象とは、実施された研究との因果関係の有無を問わず、研究対象者に生じた全ての好ましくない又は意図しない傷病もしくはその徴候（臨床検査値の異常を含む。）とする。

研究担当者は、有害事象を認めたときは、直ちに適切な処置を行うとともに、診療録等に記載する。また、試験薬の投与を中止した場合や、有害事象に対する治療が必要となった場合には、研究対象者にその旨を伝える。

（2）重篤な有害事象の報告

重篤な有害事象は、次の通りに定義する。

1）死に至るもの

2）生命を脅かすもの

3）治療のための入院又は入院期間の延長が必要となるもの

4）永続的又は顕著な障害・機能不全に陥るもの

5）子孫に先天異常を来すもの

研究担当者は、重篤な有害事象の発生を知った場合には、研究対象者等への説明等、必要な措置を講じるとともに、速やかに研究責任者に報告する。

研究責任者は、侵襲を伴う研究の実施において重篤な有害事象の発生を知った場合には、速やかに、その旨を研究機関の長に報告するとともに、適切な対応を図り、また、速やかに当該研究の実施に携わる研究担当者等に対して、当該有害事象の発生に係る情報を共有する。

（3）重要な有害事象の報告

該当なし

（4）その他の有害事象

その他の有害事象については、研究担当者は適切に診療録等に記載する。

**13．研究実施計画書等の承認・変更、改訂**

研究責任者は、予め臨床研究計画書等を研究機関の長へ提出し、研究の実施に関して自主臨床研究審査委員会（以下、審査委員会）の承認及び研究機関の長の許可を得る。また、研究実施計画書等の変更又は改訂を行う場合は、速やかに定められた作業手順にしたがって研究機関の長に改訂版を提出し、審査委員会の承認及び、研究機関の長の許可を得る。

**14．研究の中止・中断、終了**

（1）研究の中止、中断

研究担当者は、以下の事項に該当する場合は、研究実施継続の可否を検討する。

- - 1. 安全性及び有効性に関する事項及びその他の重要な情報を知りえたとき。
    2. 研究対象者の組み入れが困難で、予定症例数に達することが極めて困難であると判断されたとき。
    3. 予定症例数又は予定期間に達する前に、研究の目的が達成されたとき。
    4. 審査委員会により、実施計画等の変更の指示があり、これを受入れることが困難と判断されたとき。

研究責任者は、審査委員会により中止の勧告あるいは指示があった場合は、研究を中止する。また、研究の中止又は中断を決定した時は、速やかに研究機関の長にその理由とともに文書で報告する。

（2）研究の終了

研究の終了時には、研究責任者は速やかに研究終了報告書を研究機関の長に提出する。

**15．研究実施期間**

実施許可日～平成30年3月31日

**16．目標症例数とその設定根拠及び統計解析方法**

（1）目標症例数とその設定根拠

＜研究１＞

24例（ボランティア7例、糖尿病患者17例）

【設定根拠】

相関係数0.6と仮定し、検出力80%、有意水準5%で設定するとn=19例が必要となる。脱落症例を2割と想定してn=24で設定した。

＜研究２＞

11例（ボランティア6例、糖尿病患者5例）

【設定根拠】

2回測定における期待級内相関係数を0.8とし帰無仮説における級内相関係数を0.1とすると、有意水準5%、検出力80%で必要な対象者数は9例となる^6^⁾。脱落症例を2割と想定してn=11で設定した。

（2）統計解析方法

研究1については、SITTの結果より算出したKitt(SITT)と簡易インスリン負荷試験より算出したKitt(i.m.)の相関性に関してPearsonの相関係数を算出し、相関係数0を帰無仮説とした両側検定を行う。較正直線の推定については、分散比1の直交回帰を行う。

研究2については、級内相関係数に対してFisherのZ変換を行い、帰無仮説0.1に対して片側2.5%で検定を行う^6^⁾。

その他の、統計解析に関しては、統計解析計画書に記載する。

**17．研究対象者の人権に対する配慮**

本研究のすべての担当者は、「ヘルシンキ宣言（2013年10月修正）」及び「人を対象とする医学系研究に関する倫理指針（平成26年文部科学省・厚生労働省告示第3号）」を遵守して実施する。

**18．個人情報の取扱い**

研究実施に係る試料・情報を取扱う際は、予め研究対象者の個人情報とは無関係の番号を付して連結可能匿名化として管理し、研究対象者の秘密保護に十分配慮する。匿名化対応表は、研究責任者が厳重に保管するよう監督する。また、研究責任者等が本研究で得られた情報を公表する際は、研究対象者を特定できる情報を含まないようにする。

**19．同意取得方法**

研究担当者は、審査委員会で承認の得られた同意説明文書を研究対象者に渡し、文書及び口頭による十分な説明を行い、研究対象者の自由意思による同意を文書で取得する。

研究担当者は、研究対象者の同意に影響を及ぼす情報が得られたときや、研究対象者の同意に影響を及ぼすような実施計画等の変更が行われるときは、速やかに研究対象者に情報提供し、研究に参加するか否かについて研究対象者の意思を予め確認するとともに、事前に審査委員会の承認を得て同意説明文書等の改訂を行い、研究対象者の再同意を得ることとする。

1. 研究の名称及び当該研究の実施について研究機関の長の許可を受けている旨
2. 研究機関の名称及び研究責任者の氏名
3. 研究の目的及び意義
4. 研究の方法（研究対象者から取得された試料・情報の利用目的を含む。）及び期間
5. 研究対象者として選定された理由
6. 研究対象者に生じる負担並びに予測されるリスク及び利益
7. 研究が実施又は継続されることに同意した場合であっても随時これを撤回できる旨（研究対象者等からの撤回の内容に従った措置を講じることが困難となる場合があるときは、その旨及びその理由）
8. 研究が実施又は継続されることに同意しないこと又は同意を撤回することによって研究対象者等が不利益な取扱いを受けない旨
9. 研究に関する情報公開の方法
10. 研究対象者等の求めに応じて、他の研究対象者等の個人情報等の保護及び当該研究の独創性の確保に支障がない範囲内で研究計画書及び研究の方法に関する資料を入手又は閲覧できる旨並びにその入手又は閲覧の方法
11. 個人情報等の取扱い（匿名化する場合にはその方法を含む。）
12. 試料・情報の保管及び廃棄の方法
13. 研究の資金源等、研究機関の研究に係る利益相反及び個人の収益等、研究者等の研究に係る利益相反に関する状況
14. 研究対象者等及びその関係者からの相談等への対応
15. 研究対象者等に経済的負担又は謝礼がある場合には、その旨及びその内容（本研究は該当しない）
16. 通常の診療を超える医療行為を伴う研究の場合には、他の治療方法等に関する事項（本研究は該当しない）
17. 通常の診療を超える医療行為を伴う研究の場合には、研究対象者への研究実施後における医療の提供に関する対応（本研究は該当しない）
18. 研究の実施に伴い、研究対象者の健康、子孫に受け継がれ得る遺伝的特徴等に関する重要な知見が得られる可能性がある場合には、研究対象者に係る研究結果（偶発的所見を含む。）の取扱い
19. 侵襲を伴う研究の場合には、当該研究によって生じた健康被害に対する補償の有無及びその内容
20. 研究対象者から取得された試料・情報について、研究対象者等から同意を受ける時点では特定されない将来の研究のために用いられる可能性又は他の研究機関に提供する可能性がある場合には、その旨と同意を受ける時点において想定される内容

㉑　研究対象者の秘密が保全されることを前提として、モニタリングに従事する者及び審査委員会が、必要な範囲内において当該研究対象者に関する試料・情報を閲覧する旨

**20．研究対象者の健康被害への対応と補償**

（研究対象者が患者の場合）本研究の実施に伴い、研究対象者に健康被害が発生した場合は、研究担当者は適切な処置を講じる。また、健康被害に対する補償は、「人を対象とする医学系研究に関する倫理指針」に従って行う。すなわち、本研究に起因して発生した死亡又は後遺障害（障害等級一級及び二級）に対し、補償金を準備する。これ以外の健康被害に対しては、研究対象者の保険診療内で検査や治療等、必要な処置を行う。

（研究対象者が健常者の場合）本研究の実施に伴い、研究対象者に健康被害が発生した場合は、研究担当者は適切な処置を講じる。また、健康被害に対する補償は、「人を対象とする医学系研究に関する倫理指針」に従って行う。すなわち、本研究に起因して発生した死亡又は後遺障害（障害等級一級から十四級）に対し、補償金を準備する。これ以外の健康被害に対しては、研究対象者の保険診療内で検査や治療等、必要な処置を行う。

**21．研究機関の長への報告内容及び方法**

（1）進捗状況等の報告

研究責任者は、少なくとも年に1回、研究の進捗状況及び研究の実施に伴う有害事象の発生状況を研究機関の長に文書で報告する。

（2）重篤な有害事象の報告

研究責任者は、重篤な有害事象の発生を知った場合には、速やかにその旨を研究機関の長に報告する。

（3）研究の倫理的妥当性若しくは科学的合理性を損なう事実等の情報を得た場合

研究責任者は、研究の倫理的妥当性若しくは科学的合理性を損なう事実若しくは情報又は損なうおそれのある情報であって研究の継続に影響を与えると考えられるものを得た場合には、遅滞なくその旨を研究機関の長に報告する。

（4）研究の実施の適正性若しくは研究結果の信頼を損なう事実等の情報を得た場合

研究担当者は、研究の実施の適正性若しくは研究結果の信頼を損なう事実若しくは情報又は損なうおそれのある情報を得た場合には、速やかにその旨を研究機関の長に報告する。

（5）研究終了（中止の場合を含む、以下同じ。）の報告

研究責任者は、研究を終了したときは、14に従ってその旨及び研究の結果概要を文書により遅滞なくその旨を研究機関の長に報告する。

（6）研究に用いる試料及び情報の管理状況

研究責任者は、人体から取得された試料及び情報等の保管について、24.(1)(2)に従って必要な管理を行い、管理状況について研究機関の長に報告する。

（7）研究結果の公表の報告

研究責任者は、結果の最終の公表を行ったときは、25に従って遅滞なく研究機関の長へ報告する。また、最終の公表を行ったとして報告した後に、研究結果の公表を行うこととなった場合は、速やかにその旨を研究機関の長に報告する。

**22．効果・安全性評価委員会**

　該当なし

**23．研究対象者の費用負担**

本研究で用いる医薬品の投与及び実施する検査（空腹時血糖値、インスリン、S-Cペプチド、血漿アミノ酸濃度、アディポネクチン、TNF-α、レプチン、SITTの血糖値、簡易インスリン負荷試験の血糖値）は研究責任者が所属する診療科の研究費で賄う。それ以外は通常の保険診療内で行われるため、研究に参加することによる研究対象者の費用負担は発生しない。ボランティアに関しても研究に参加することによる研究対象者の費用負担は発生しない。

　尚、研究対象者へ謝礼等の支給はおこなわない。

**24．試料・情報等の保管及び廃棄の方法**

（1）試料の保管及び廃棄の方法

研究責任者は、定めたれた保管方法に従って研究担当者等が適切に保管するよう指導し、試料の漏えい、混交、盗難、紛失等が起こらないよう必要な管理を行う。

採取した血液（測定後の残余分、保管用採血分）は、研究対象者の同意が得られれば研究終了後5年後まで内科Ⅰ医局で冷凍保管する。研究対象者の同意が得られない場合には、測定後即廃棄する。

廃棄する際は、匿名化し個人情報に注意して行う。

（2）情報等の保管及び廃棄の方法

研究責任者は、定めたれた保管方法に従って研究担当者等が情報等（研究に用いられる情報及び当該情報に係る資料）を正確なものにするよう指導し、情報等の漏えい、混交、盗難、紛失等が起こらないよう必要な管理を行う。

本研究で得られた情報等は、医局内内科Ⅰ病棟内の施錠ができるキャビネットに保管する。

研究責任者は、研究に用いられる情報等については、可能な限り長期間保管し、少なくとも、当該研究の終了について報告された日から5年を経過した日又は当該研究の結果の最終の公表について報告された日から3年を経過した日のいずれか遅い日までの期間、適切に保管する。また、連結可能匿名化された情報について、本院が対応表を保有する場合には、対応表の保管についても同様とする。

廃棄する際は、匿名化し個人情報に注意して行う。

（3）試料及び情報の二次利用について

本研究で得られた研究対象者の試料・情報は、同意を受ける時点では特定されない将来の研究のために用いる可能性がある。その場合には、別途研究対象者に説明した上で実施する。

**25．研究に関する情報公開の方法及び研究結果の公表**

研究責任者は、公開データベースに当該研究の概要をその実施に先立って登録し、研究計画書の変更及び研究の進捗に応じて適宜更新する。研究を終了したときは、遅滞なく、当該研究の結果を登録する。また、結果を公表する際は、研究対象者等及びその関係者の人権又は研究担当者等及びその関係者の権利利益の保護のために必要な措置を講じた上で行う。結果の最終の公表を行ったときは、遅滞なく研究機関の長へ報告する。

登録する公開データベースは、大学病院医療情報ネットワーク（UMIN-CTR）とする。

**26.　知的財産権の帰属について**

この研究から成果が得られ、知的財産権などが生じる場合は、その権利は当研究グループに帰属する。

**27．研究資金及び利益相反**

本研究は、研究責任者が所属する診療科の研究費で実施する。また、本研究の研究担当者は、「北海道大学病院における臨床研究に係る利益相反マネジメント内規」の規定にしたがって、利益相反審査委員会に必要事項を申告し、その審査と承認を得るものとする。

**28．モニタリング**

研究責任者は、研究の信頼性の確保に努め、研究対象者の人権、安全および福祉が保護されていること、本研究が研究実施計画書を遵守して実施されていること、および研究担当者から報告されたデータが正確に収集されていることを確認することを目的としてモニタリングを実施する。研究責任者は、適切にモニタリングが行われるようモニタリング担当者を指名するものとする。モニタリング担当者は、予め作成されたモニタリング手順書に従ってモニタリングを実施する。

**29．監査**

本研究では、モニタリングにより品質管理を適正に実施することとし、監査は行わない。

**30．研究実施体制**

本研究は以下の体制で実施する。

【研究責任者】

渡部　拓　 （北海道大学病院内科Ⅰ・助教）

011-706-5911（内線 35911）

＊研究分担者・協力者については様式A-2自主臨床研究分担者・協力者リスト」を参照

【連絡先】

内科Ⅰ 医局 011-706-5911（内線 35911）

同　　 病棟 011-706-5808（内線 35808）

同 外来 011-706-5752（内線 35752）

【相談窓口】

　　　　　内科Ⅰ　　　　　　　　病棟 　011-706-5808（内線 35808）

【データマネジメント実施施設】

林下　晶子　　（北海道大学病院内科Ⅰ・医員）

e-mail : akiko.hayashishita@pop.med.hokudai.ac.jp

PHS 82377

【研究事務局】

北海道大学　内科Ⅰ

連絡先：〒060-0838 北海道札幌市北区北15条西7丁目

北海道大学病院　内科Ⅰ　医局　011-706-5911

【統計解析責任者】

伊藤　陽一　　（北海道大学　医学研究科　社会医学講座）

【モニタリング実施施設】

　　　　鈴木　雅　　　（北海道大学病院内科Ⅰ・助教）

011-706-5911（内線 35911）

　　【登録事務局】

　　　　株式会社エクサム

　　　　〒060-0001　札幌市中央区北1条西5丁目2

TEL：011-222-5225　　FAX：011-222-5265　　【割付担当者】

　　　　中島　圭吾　　　(株式会社エクサム)

　　　　　　　　　　　　011-222-5225

【外部支援機関】

株式会社エスアールエル　北海道ラボラトリー

〒064-0919札幌市中央区南19条西13丁目2番25号

TEL：011-511-9991　　FAX：011-520-8161

業務委託契約に基づき、研究参加施設から試料を回収し、測定を行う。

**31．参考資料・文献リスト**

1. 糖尿病専門医研修ガイドブック 改定第6版. 日本糖尿病学会編. 診断と治療社
2. Matthews DR et al: Diabetologia. 1985; 28: 412-419
3. Rabinowitz D et al: J Clin Invest. 1962; 41: 2173-2181
4. Bonora E et al: J Clin Endocrinol Metab. 1989; 68: 374-378
5. Lundbaek K et al: British Medical Journal. 1962; J 2: 1507-1513
6. Machin D et al: Sample size tables for clinical studies. 3^rd^ ed. 2009. Wiley-Blackwell.
